# Stimulating the Brain Makes the Fingers More Sensitive

**DOI:** 10.1371/journal.pbio.0030408

**Published:** 2005-10-18

Repetitive transcranial magnetic stimulation (rTMS) has more than a whiff of Buck Rogers to it: a magnetic wand passes over the surface of the skull, triggering changes in the brain underneath. But it's not science fiction, and in the past decade, rTMS has emerged as an intriguing technique for exploring brain function, and a promising, though still unproven, form of therapy. In this issue, Hubert Dinse, Martin Tegenthoff, and their colleagues show that a short course of rTMS can increase finger sensitivity for up to two hours after treatment ends, and that this change corresponds to an increase in the size of the brain map representing the finger.

rTMS is applied with an electromagnetic coil in the shape of a figure-eight, placed on the scalp directly over the targeted portion of the brain. Short bursts of a strong magnetic pulse stimulate electrical currents within. Sensory input from each region of the body is represented on the surface of the brain, and the location of any region—in this case, the right index finger—can be mapped to allow precise targeting of the rTMS. The authors adjusted the strength of the magnetic field to just below that which triggered a sensory response in the finger, and then applied intermittent pulses of stimulation over the course of about ten minutes.

They tested the sensitivity of the index finger by determining how far apart two simultaneously applied pinpricks needed to be for the subject to distinguish them as separate stimuli. rTMS increased this two-point discrimination by about 15% immediately after stimulation, an effect that gradually diminished but still remained significant over the course of the next two hours. The effect was fairly specific for the right index finger: there was no effect on the left index finger, which is represented in the opposite hemisphere, and only a small effect on the right ring finger, which is represented several millimeters away from the index finger in the same hemisphere. When stimulation was applied over the area representing the lower leg, the index finger did not become more sensitive. [Fig pbio-0030408-g001]

**Figure pbio-0030408-g001:**
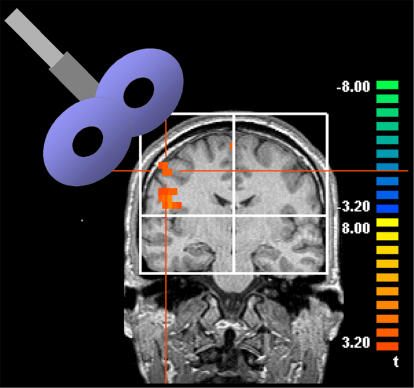
Transcranial magnetic stimulation alters sensory perception and activity in sensory cortical areas

The authors used functional magnetic resonance imaging (fMRI) to see how the brain changed in response to the stimulation. They found that the region representing the index finger got larger, and that the degree of increase in any one subject corresponded to the degree of increased sensitivity in that same subject. As the sensory effect faded, so too did the fMRI changes. Thus, the cortex itself undergoes changes as a result of rTMS.

Practice affects the brain and the brain affects practice—it now appears possible to directly intervene in this brain–behavior loop to improve short-term tactile performance. Other recent work by the same authors shows that rTMS can also improve visual discrimination, suggesting a potential for affecting changes throughout the brain. These results are unlikely to be of immediate benefit to those who rely on exquisite sensitivity in their fingers, whether surgeons or safecrackers, as the equipment needed for rTMS is cumbersome and the duration of the effect relatively short. However, a related technique, transcranial direct current stimulation, employs much more portable equipment, suggesting it may find a role in neurorehabilitation. Further study of both techniques will be needed to determine the future of this futuristic technology. —*Richard Robinson*

